# Different risk and prognostic factors for liver metastasis of breast cancer patients with *de novo* and relapsed distant metastasis in a Chinese population

**DOI:** 10.3389/fonc.2023.1102853

**Published:** 2023-04-14

**Authors:** Ningning Zhang, Yimei Xiang, Qing Shao, Jing Wu, Yumin Liu, Hua Long, Dan Tao, Xiaohua Zeng

**Affiliations:** ^1^ Department of Breast Cancer Center, Chongqing University Cancer Hospital, Chongqing, China; ^2^ Department of Breast Cancer Center, Chongqing University Cancer Hospital, School of Medicine, Chongqing University, Chongqing, China; ^3^ Department of Medical Record, Chongqing University Cancer Hospital, Chongqing, China; ^4^ Department of Radiation Oncology, Chongqing University Cancer Hospital, Chongqing, China; ^5^ Chongqing Key Laboratory of Translational Research for Cancer Metastasis and Individualized Treatment, Chongqing University Cancer Hospital, Chongqing, China; ^6^ Chongqing Key Laboratory for Intelligent Oncology in Breast Cancer (iCQBC), Chongqing University Cancer Hospital, Chongqing, China

**Keywords:** breast cancer, risk factors, liver metastasis, *de novo* distant metastasis, relapsed distant metastasis, prognosis

## Abstract

**Purpose:**

The present study aimed to identify clinicopathological characteristics of breast cancer liver metastasis (BCLM) as well as to characterize the risk and prognostic factors for the liver metastasis (LM) of breast cancer patients with *de novo* and relapsed distant metastasis in a Chinese population.

**Materials and methods:**

Patients with metastatic breast cancer (MBC) who were hospitalized in the Breast Cancer Center at Chongqing University between January 2011 and December 2019 were included in the present study. Logistic regression analyses were conducted to identify risk factors for the presence of BCLM. Cox proportional hazard regression models were performed to determine the prognostic factors for the survival of BCLM patients. The correlation between LM and overall survival was assessed by the Kaplan–Meier method.

**Results:**

In total, 1,228 eligible MBC patients, including 325 cases (26.5%) with *de novo* metastasis (cohort A) and 903 cases (73.5%) with relapsed metastasis (cohort B), were enrolled in the present study. In cohort A and cohort B, 81 (24.9%) and 226 (25.0%) patients had BCLM, respectively. Patients in these two cohorts had different clinicopathological features. Logistic regression analysis identified that the human epidermal growth factor receptor 2 (HER2) status in cohort A as well as the HER2 status and invasive ductal carcinoma histology in cohort B were risk factors for BCLM. The median OS of patients with LM was inferior to that of non-LM patients (17.1 *vs.* 37.7 months, *P* = 0.0004 and 47.6 *vs.* 84.0 months, *P* < 0.0001, respectively). Cox analysis identified that the primary T stage, Ki67 level, and breast surgery history were independent prognostic factors for cohorts A and B, respectively.

**Conclusions:**

*De novo* and relapsed MBC patients have different risk and prognostic factors for LM. Patients with BCLM have an unfavorable prognosis.

## Introduction

Breast cancer is the most common type of cancer worldwide (1). At the time of breast cancer diagnosis, approximately 5%–15% of patients have distant metastases (*de novo* metastatic disease) (2, 3). There are 20%–30% of early breast cancer patients who experience recurrence or relapse with distant metastases, such as bone, lung, liver, and/or brain metastases, after standard initial treatment (4, 5). Metastatic breast cancer (MBC) is incurable, and almost all fatalities due to breast cancer are caused by distant metastases (6–11).

It is worth noting that the liver is the third most common metastatic organ. Patients with breast cancer liver metastasis (BCLM) have a worse prognosis than those with bone or other organ metastases with 5-year survival rates of only 3.8%–12% (12), and BCLM patients have a median survival time of 2–3 years (6, 13). In addition, more than 20% of deaths from breast cancer are caused by liver metastasis (LM) (14, 15). BCLM is often asymptomatic or presents atypical symptoms, which tend to be ignored in the beginning stage of LM. Moreover, most patients with BLCM have extensive LM at the time of discovery as well as bone, lung, brain, or other organ metastases (16). Because every breast cancer patient has the risk of developing LM, it is important to identify the risk factors associated with the occurrence of BCLM to develop appropriate individualized treatment schemes to prolong survival time (17–21).

Breast cancer is a heterogeneous disease with multiple histological and molecular profiles related to different prognoses. Previous studies have demonstrated the association between clinicopathological factors and the predisposition to BCLM (6, 22). However, those studies did not differentiate *de novo* and recurrent distant metastasis in breast cancer patients. Many previous studies have indicated that patients with *de novo* MBC represent a distinct population of patients with recurrent MBC; changes in the tumor phenotype have been found between primary and recurrent breast cancer (3, 23–25). Their results suggest that *de novo* and relapsed MBC patients are distinct. Further, studies focusing on LM in Chinese individuals with breast cancer are lacking.

In the present study, we aimed to identify the clinicopathological characteristics of BCLM and explore the risk factors that affect the incidence and prognosis of LM in patients with *de novo* MBC and patients with relapsed MBC in the Chinese population.

## Materials and methods

### Patient selection

Between January 2011 and December 2019, patients with histologically proven breast cancer who were admitted to the Breast Cancer Center of Chongqing University Cancer Hospital (Chongqing, China) were screened. This cancer center is one of the largest in southwest China, covering an approximately 82,402.95 km^2^ area with a population of 32.05 million residents. The inclusion criteria were as follows: female patients with histologically diagnosed breast cancer with at least one distant site metastasis and with complete information on molecular typing. The exclusion criteria were as follows: patients with bilateral breast cancer; patients with other tumor disorders; patients with recurrence only in the regional lymph node and/or chest wall; and patients with unqualified medical records. This study was conducted in accordance with the Declaration of Helsinki and the principles of Good Clinical Practice, and it was approved by the Medical Ethics Committee of the Chongqing University Cancer Hospital (CZLS2022177-A).

### Data collection

The following data were extracted from patient medical records: demographic information; clinicopathological factors, including age, menopause, and menstrual and reproductive history; hepatitis B infection information; the date of the breast cancer diagnosis; the date and location of first organ metastasis; height and weight at the diagnosis with distant metastasis; T, N, and M stages; histologic type and grades; estrogen receptor (ER), progesterone receptor (PR), and human epidermal growth factor receptor 2 (HER2) status; and treatment information. The body mass index (BMI) was subtyped based on the criteria of the World Health Organization (WHO) as follows: underweight (<18.5 kg/m^2^), normal weight (18.5–24.9 kg/m^2^), overweight (24.9–29.9 kg/m^2^), and obese (≥30 kg/m^2^). Menarche was defined as early menarche (≤12 years), normal menarche (13–14 years), and late menarche (≥15 years). The diagnosing and staging of breast cancer were in line with the tumor–node–metastasis (TNM) system based on the guidelines of the 7th Edition of American Joint Committee on Cancer (AJCC) (26). The WHO international histological classification was used to assess the final histopathological diagnosis (27). The primary tumor’s histologic grade was determined using the modified Scarff–Bloom–Richardson system (mSBR) (28). ER and PR were defined as positive if more than 1% of tumor cells showed positive nuclear staining by immunohistochemistry (IHC). IHC 3+ staining and IHC 2+ staining or ambiguous fluorescence *in situ* hybridization (FISH) results were regarded as positive. Some individuals with HER2 IHC 2+ but no FISH results were defined as HER2 negative.

The time to diagnose distant organ metastasis, including the bone, lung/pleura, liver, and central nervous system (CNS), was recorded. CNS metastatic patients were defined as those with either parenchymal brain metastasis and/or leptomeningeal metastasis. The evidence of organ metastasis was defined as the presence of clinical manifestations of metastases and confirmed with imaging/pathology examination and/or radiologic changes using a computed tomography scan with contrast or whole-body bone scan. Oligometastasis was defined as only one organ metastasis, and polymetastasis was defined as ≥2 organ metastasis, including LM.

### Follow-up information

All patients were routinely followed up by telephone, outpatient visits, or hospitalization information. During the first 6 months after surgery, drug treatment, or radiotherapy, follow-up visits were scheduled at least once a month and every 6 months after that unless there were any unscheduled complications. Patient survival outcome information was collected through active and passive follow-ups. The fundamental cause of death was derived from medical records or information from immediate family members. The date of the last follow-up was 17 September 2021. Metastasis-free survival (MFS) was defined as the time interval from the breast cancer diagnosis to the first detection of distant metastasis, including LM, lung/pleura metastasis, bone metastasis, and/or CNS metastasis. Overall survival (OS) was calculated from the date of diagnosis with primary breast cancer to the date of death or last follow-up.

### Statistical analyses

The Pearson chi-square test, Fisher’s exact test, or Kruskal–Wallis test was used to examine the associations between LM and clinicopathologic parameters as appropriate. Multivariable logistic and Cox proportional hazard regression analyses were performed to explore risk and prognostic factors for BCLM based on the univariable regression results (*P* < 0.1). All confidence intervals (CIs) are shown at the 95% confidence level. The Kaplan–Meier method was used to plot survival curves, and the log-rank test was performed to compare the survival differences. Statistical analyses were performed using R software (version 3.4, http://www.r-project.org). A two-sided *P*-value of 0.05 or less was considered statistically significant.

## Results

### Patient characteristics

**Figure 1:**
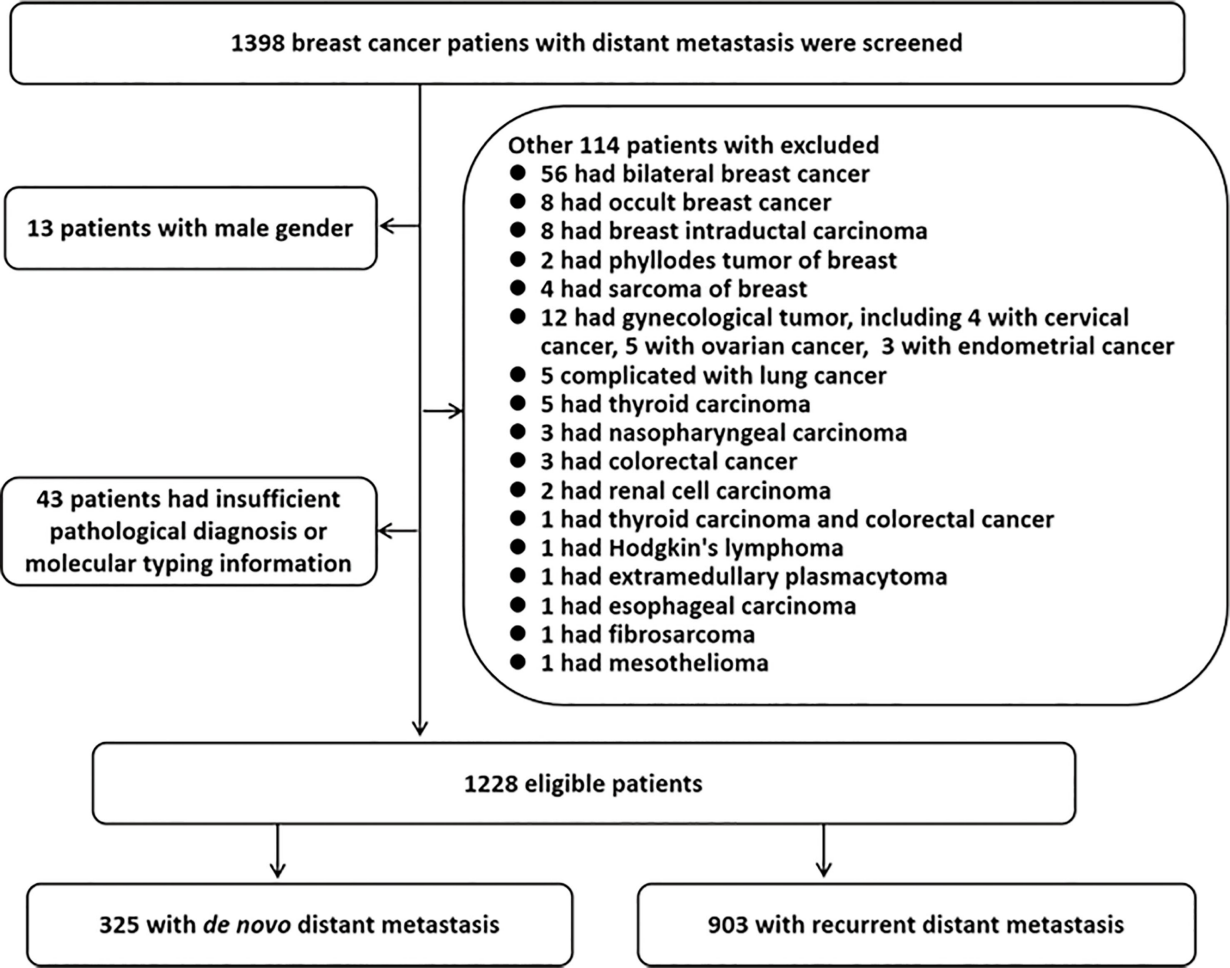
Flowchart of the patient selection progress.

In total, 1,398 MBC patients were further screened. Of these, the following patients were excluded: 13 cases were excluded due to the male gender; 43 cases were eliminated due to incomplete information on the pathological diagnosis or molecular typing; and 114 cases were eliminated due to the presence of other primary cancers. Finally, 1,228 eligible patients were enrolled in the present study, including 325 (26.5%) patients with distant metastasis at diagnosis (termed cohort A), and 903 (73.5%) patients with relapsed distant metastasis (termed cohort B). A flowchart of the patient selection process is shown in **Figure 1**.

Hormone receptor (HR) positive and HER2 negative (HR+/HER2−) was the most common subtype among the MBC patients in both cohorts A and B followed by the HER2-positive and triple-negative breast cancer (TNBC) subtypes. The frequency of LM was 24.9% (81/325) and 25.0% (226/903) in cohorts A and B, respectively. The other sites of metastasis were the bone, lung/pleura, and CNS with percentages of 51.4% (167/325), 37.8% (123/325), and 9.8% (32/325) in cohort A as well as 46.3% (418/903), 42.8% (387/903), and 7.8% (71/903) in cohort B, respectively. The clinicopathological features of the patients stratified by LM in the two cohorts are summarized in **Table 1**. The mean age at diagnosis of breast cancer was 52.1 and 48.9 years in cohorts A and B, respectively (*P* < 0.0001), but no significant difference in age was found between the two cohorts (52.2 *vs.* 51.9 years). The mean age of patients with LM at the time of diagnosis of breast cancer in cohort A was older than that in cohort B (*P* = 0.026). The mean age at diagnosis of MBC in patients with LM was younger than that in patients without LM, but a significant difference was found only in cohort B (*P* = 0.015). In cohort B, patients with invasive ductal histology were more likely to have LM (*P* = 0.003). Patients with late menarche and the ER+ status tended to have a lower risk for LM, but there was no statistical difference. Regardless whether patients had *de novo* MBC or relapsed MBC after previous treatment, LM was more likely to occur in HER2+ tumors (*P* < 0.0001). Compared to patients in cohort A, patients in cohort B had lower T (*P* < 0.0001) and N staging (*P* = 0.011). Patients with LM in cohort A had a higher risk for CNS metastasis than those in cohort B (*P* = 0.015). Compared to patients without LM, the proportion of patients with distant lymph node metastasis and bone metastasis were lower in patients with LM (*P* < 0.0001). Among all patients, patients with LM had a higher risk of polymetastasis (*P* < 0.0001). In cohort B, the median time of MFS in MBC patients with LM was significantly shorter than that in those without LM (21.0 months *vs.* 27.0 months, *P* = 0.001), and the proportion of patients with MFS less than 1 year was higher in BCLM patients compared to non-BCLM patients (*P* < 0.0001).

**Table 1:** Patient characteristics by the liver metastasis (LM) status in *de novo* and relapsed metastatic breast cancer.

Characteristic	Cohort A (N = 325) Patients with *de novo* distant metastasis			Cohort B (N = 903) Patients with recurrent distant metastasis			Cohort A *vs.* B (Patients with liver metastasis)
	Liver Metastasis (N=81) No. (%)	No Liver Metastasis (N=244) No. (%)	*P*-value	Liver Metastasis (N = 226) No. (%)	No Liver Metastasis (N = 677) No. (%)	*P*-value	*P*-value
Age at diagnosis (mean)	51.1	52.5	0.338	48.1	49.2	0.147	0.026
<50	40 (49.4%)	107 (43.9%)	0.386	136 (60.2%)	375 (55.4%)	0.209	0.092
≥50	41 (50.6%)	137 (56.1%)		90 (39.8%)	302 (44.6%)		
Age at metastasis (mean)	51.1	52.5	0.338	50.5	52.4	0.015	0.660
<55	58 (71.6%)	146 (59.8%)	0.058	154 (68.1%)	430 (63.5%)	0.208	0.563
≥55	23 (28.4%)	98 (40.2%)		72 (31.9%)	247 (36.5%)		
Laterality			0.771			0.986	0.958
Left	44 (54.3%)	128 (52.5%)		122 (54.0%)	365 (53.9%)		
Right	37 (45.7%)	116 (47.5%)		104 (46.0%)	312 (46.1%)		
Menopausal status			0.501			0.115	0.071
Pre-	42 (51.9%)	116 (47.5%)		143 (63.3%)	388 (57.3%)		
Post-	39 (48.1%)	128 (52.5%)		83 (36.7%)	289 (42.7%)		
Menarche (years) (≥15 *vs.* low)			0.069			0.127	0.388
≤12	26 (32.1%)	80 (32.8%)		75 (33.2%)	190 (28.1%)		
13–14	37 (45.7%)	82 (33.6%)		92 (40.7%)	269 (39.7%)		
≥15	17 (21.0%)	76 (31.1%)		59 (26.1%)	211 (31.2%)		
Unknown	1 (1.2%)	6 (2.5%)		0 (0.0%)	7 (1.0%)		
Number of gravidity			0.978			0.644	0.326
≤2	46 (56.8%)	139 (57.0%)		114 (50.4%)	353 (52.1%)		
≥3	35 (43.2%)	105 (43.0%)		112 (49.6%)	323 (47.7%)		
Unknown	0 (0.0%)	0 (0.0%)		0 (0.0%)	1 (0.1%)		
Number of parity			0.461			0.565	0.836
≤1	43 (53.1%)	118 (48.4%)		123 (54.4%)	353 (52.1%)		
≥2	38 (46.9%)	126 (51.6%)		103 (45.6%)	323 (47.7%)		
Unknown	0 (0.0%)	0 (0.0%)		0 (0.0%)	1 (0.1%)		
BMI (>25 *vs.* <25)			0.558			0.322	0.900
≤18.5	2 (2.4%)	7 (2.8%)		9 (4.0%)	25 (3.7%)		
18.5–25	51 (63.0%)	142 (58.2%)		137 (60.6%)	387 (57.2%)		
25–30	22 (27.2%)	71 (29.1%)		68 (30.1%)	219 (32.3%)		
>30	5 (6.2%)	18 (7.4%)		9 (4.0%)	36 (5.3%)		
Unknown	1 (1.2%)	6 (2.5%)		3 (1.3%)	10 (1.5%)		
Family history of cancer			0.534			0.478	0.659
Yes	15 (18.5%)	38 (15.6%)		37 (16.4%)	125 (18.5%)		
No	66 (81.5%)	206 (84.4%)		189 (83.6%)	552 (81.5%)		
HBV status			0.705			0.411	0.798
HbsAg+	6 (7.4%)	22 (9.0%)		15 (6.6%)	53 (7.8%)		
HbsAg−	70 (86.4%)	214 (87.7%)		199 (88.1%)	548 (81.0%)		
Unknown	5 (6.2%)	8 (3.3%)		12 (5.3%)	76 (11.2%)		
Histology (lobular + other *vs.* ductal)			0.386			0.003	0.530
Invasive ductal	77 (95.1%)	225 (92.2%)		220 (97.4%)	620 (91.6%)		
Invasive lobular	3 (3.7%)	11 (4.5%)		1 (0.4%)	23 (3.4%)		
Other	1 (1.2%)	8 (3.3%)		5 (2.2%)	34 (5.0%)		
ER status			0.074			0.056	0.467
Positive	41 (50.6%)	151 (61.9%)		125 (55.3%)	423 (62.5%)		
Negative	40 (49.4%)	93 (38.1%)		101 (44.7%)	254 (37.5%)		
PR status			0.233			0.139	0.733
Positive	33 (40.7%)	118 (48.4%)		97 (42.9%)	329 (48.6%)		
Negative	48 (59.3%)	126 (51.6%)		129 (57.1%)	348 (51.4%)		
HER2 status			0.000			0.000	0.298
Positive	42 (51.9%)	63 (25.8%)		102 (45.1%)	205 (30.3%)		
Negative	39 (48.1%)	181 (74.2%)		124 (54.9%)	472 (69.7%)		
Ki67^			0.176			0.561	0.833
<20%	20 (24.7)	42 (17.2)		48 (21.2)	142 (21.0)		
≥20%	57 (70.4)	182 (74.6)		146 (64.6)	386 (57.0)		
Unknown	4 (4.9)	20 (8.2)		32 (14.2)	149 (22.0)		
Molecular subtypes^*^							
HR + HER2−	29 (35.8%)	133 (54.5%)		79 (35.0%)	340 (50.2%)		
HER2 positive	42 (51.9%)	63 (25.8%)	0.000	102 (45.1%)	205 (30.3%)	0.000	0.686
TNBC	10 (12.3%)	48 (19.7%)	0.003	45 (19.9%)	132 (19.5%)	0.063	0.115
Nuclear Ggade (III *vs.* I–II)			0.647			0.751	1.000
I	1 (1.2%)	1 (0.4%)		3 (1.3%)	17 (2.5%)		
II	3 (3.7%)	38 (15.6%)		107 (47.4%)	275 (40.6%)		
III	1 (1.2%)	23 (9.4%)		43 (19.0%)	122 (18.0%)		
Unknown	76 (93.8%)	182 (74.6%)		73 (32.3%)	263 (38.9%)		
Primary T stage (T1–3 *vs.* T4)			0.477			0.624	0.000
T1	8 (9.9%)	16 (6.6%)		33 (14.6%)	85 (12.6%)		
T2	24 (29.6%)	76 (31.1%)		120 (53.1%)	329 (48.6%)		
T3	11 (13.6%)	25 (10.2%)		29 (12.8%)	80 (11.8%)		
T4	37 (45.7%)	121 (49.6%)		27 (12.0%)	65 (9.6%)		
Unknown	1 (1.2%)	6 (2.5%)		17 (7.5%)	118 (17.4%)		
Regional N stage (N0 *vs.* N1–3)			0.597			0.975	0.011
N0	10 (12.3%)	25 (10.2%)		59 (26.1%)	203 (30.0%)		
N1	14 (17.3%)	36 (14.8%)		59 (26.1%)	168 (24.8%)		
N2	20 (24.7%)	67 (27.5%)		62 (27.4%)	158 (23.3%)		
N3	37 (45.7%)	116 (47.5%)		46 (20.4%)	148 (21.9%)		
Breast surgery^#^			0.829			0.155	0.000
Breast conservation	0 (0.0%)	0 (0.0%)		208 (92.0%)	619 (91.4%)		
Radical mastectomy	0 (0.0%)	0 (0.0%)		1 (0.4%)	8 (1.2%)		
Palliative surgery	1 (1.2%)	6 (2.5%)		2 (0.8%)	21 (3.1%)		
None	80 (98.8%)	238 (97.5%)		15 (6.6%)	29 (4.3%)		
Neoadjuvant therapy			/			0.351	/
Yes	/	/		93 (41.2%)	255 (37.7%)		
No	/	/		133 (58.8%)	422 (62.3%)		
Treatment before metastasis			/				/
Chemotherapy	/	/		217 (96.0%)	633 (93.5%)	0.163	
Radiotherapy	/	/		94 (41.6%)	251 (37.0%)	0.226	
Anti-HER2 therapy	/	/		14 (13.7%)	42 (20.5%)	0.148	
Endocrine	/	/		94 (72.3%)	271 (60.2%)	0.157	
None	81 (100.0%)	244 (100.0%)		5 (2.2%)	25 (3.7%)	0.282	
Distant lymph node metastasis			0.189			0.009	0.309
Yes	24 (29.6)	92 (37.7)		54 (23.9)	225 (33.2)		
No	57 (70.4)	152 (62.3)		172 (76.1)	452 (66.8)		
Bone metastasis			0.501			0.000	0.061
Yes	39 (48.1%)	128 (52.5%)		82 (36.3%)	336 (49.6%)		
No	42 (51.9%)	116 (47.5%)		144 (63.7%)	341 (50.4ta%)		
Lung/Pleura metastasis			0.662			0.894	0.294
Yes	29 (35.8%)	94 (38.5%)		96 (42.5%)	291 (43.0%)		
No	52 (64.2%)	150 (61.5%)		130 (57.5%)	386 (57.0%)		
CNS metastasis			0.193			0.100	0.015
Yes	11(13.6%)	21 (8.6%)		12 (5.3%)	59 (8.7%)		
No	70(86.4%)	223 (91.4%)		214 (94.7%)	618 (91.3%)		
Metastasis status			0.000			0.000	0.419
Oligometastasis	25 (30.9%)	171 (70.1%)		81 (35.8%)	467 (69.0%)		
Polymetastasis	56 (69.1%)	73 (29.9%)		145 (64.2%)	210 (31.0%)		
MFS (years)						0.000	/
≤1	/	/		81 (35.8%)	144 (21.3%)		
>1	/	/		145 (64.2%)	533 (78.7%)		

BMI, body mass index; TNBC, triple-negative breast cancer; CNS, central nervous system; MFS, metastasis-free survival. *, HER2 positive vs. HR + HER2− and TNBC vs. HR+HER2−; #, surgery vs. none; ^, Ki67≥20% vs. Ki67 <20%.

### Univariable and multivariable logistic regression analyses for breast cancer liver metastasis presence

The univariable logistic regression analysis results for the presence of LM in cohorts A and B are shown in **Table 2**. In cohort A, patients with a younger age at the diagnosis of MBC (≥55 *vs.* <55 years, OR = 0.591, 95% CI = 0.342–1.020, *P* = 0.059), ER− status (positive *vs.* negative, OR = 0.631, 95% CI = 0.380–1.048, *P* = 0.075), and HER2+ status (positive *vs.* negative, OR = 3.094, 95% CI = 1.836–5.213, *P* = 0.000) were more likely to present with LM. In cohort B, patients with the ER− status (positive *vs.* negative, OR = 0.743, 95% CI = 0.548–1.008, *P* = 0.056), HER2+ status (positive *vs.* negative, OR = 1.907, 95% CI = 1.400–2.598, *P* = 0.000), HbsAg− status (HbsAg + *vs.* HbsAg−, OR = 0.681, 95% CI = 0.513–0.903, *P* = 0.008) and invasive ductal carcinoma (IDC) histology (invasive lobular + other *vs.* invasive ductal, OR = 0.297, 95% CI = 0.126-0.698, *P* = 0.005) had a higher risk to present with LM. Multivariable logistic regression analyses indicated that the HER2 status (positive *vs.* negative, OR = 2.845, 95% CI = 1.618–5.000, *P* = 0.000) in cohort A as well as HER2 status (positive *vs.* negative, OR = 1.689, 95% CI = 1.217–2.343, *P* = 0.001) and histology (invasive lobular + other *vs.* invasive ductal, OR = 0.349, 95% CI = 0.147–0.831, *P* = 0.017) in cohort B were independent predictors for the presence of BCLM (**Table 3**).

**Table 2:** Univariable logistic regression analysis for LM in breast cancer patients with *de novo* and recurrent distant metastasis.

Variable	Cohort A (N = 325) Patients with *de novo* distant metastasis			Cohort B (N = 903) Patients with recurrent distant metastasis		
	OR	95% CI	*P-*value	OR	95% CI	*P*-value
Age at diagnosis (≥50 *vs.* <50 years)	0.801	0.484–1.325	0.387	0.822	0.605–1.116	0.209
Age at metastasis (≥55 *vs.* <55 years)	0.591	0.342–1.020	0.059	0.814	0.591–1.122	0.208
Laterality (right *vs.* left)	0.928	0.560–1.536	0.771	0.997	0.737–1.349	0.986
Menopausal status (post- *vs.* pre-)	0.842	0.509–1.392	0.501	0.779	0.571–1.063	0.115
Menarche (≥15 *vs.* ≤14 years)	0.679	0.398–1.157	0.155	0.832	0.601–1.151	0.266
Number of gravidity (≥3 *vs.* ≤2)	1.007	0.606–1.673	0.978	1.074	0.794–1.451	0.644
Number of parity (≥2 *vs.* ≤1)	0.828	0.500–1.269	0.461	0.915	0.677–1.238	0.565
BMI (>25 *vs.* ≤25)	0.930	0.571–1.1516	0.772	0.874	0.648–1.178	0.377
Family history of cancer (yes *vs.* no)	1.232	0.638–2.382	0.535	0.865	0.578–1.293	0.478
HBV status (HbsAg+ *vs.* HbsAg−)	1.198	0.720–1.995	0.487	0.681	0.513–0.903	0.008
Histology (invasive lobular + other *vs.* invasive ductal)	0.615	0.203–1.864	0.390	0.297	0.126–0.698	0.005
ER status (positive *vs.* negative)	0.631	0.380–1.048	0.075	0.743	0.548–1.008	0.056
PR status (positive *vs.* negative)	0.734	0.441–1.222	0.234	0.795	0.587–1.077	0.139
HER2 status (positive *vs.* negative)	3.094	1.836–5.213	0.000	1.907	1.400–2.598	0.000
Ki67 (Ki67≥20% *vs.* Ki67 <20%)	0.658	0.357–1.210	0.178	1.119	0.766–1.634	0.561
Molecular subtypes						
HER2 positive *vs.* HR + HER2−	3.057	1.746–5.354	0.000	2.152	1.530–3.027	0.000
TNBC *vs.* HR + HER2−	0.955	0.433–2.107	0.910	1.456	0.959–2.210	0.078
Nuclear grade (III *vs.* I–II)	0.424	0.045–4.026	0.455	0.936	0.620–1.411	0.751
Primary T stage						
T4 *vs.* T1–3	0.832	0.501–1.382	0.478	1.127	0.698–1.822	0.624
Regional N stage (N1–3 *vs.* N0)	0.811	0.371–1.769	0.598	1.212	0.863–1.702	0.266
Breast surgery	/	/	/			
Breast surgery *vs.* none	/	/	/	0.630	0.331–1.197	0.158
Radical mastectomy *vs.* none	/	/	/	0.242	0.028–2.117	0.200
Breast conservation *vs.* none	/	/	/	0.650	0.342–1.236	0.188
Local lumpectomy *vs.* none	/	/	/	0.242	0.028–2.117	0.200
Palliative surgery *vs.* none	/	/	/	0.149	0.018–1.248	0.079
Breast conservation + radical mastectomy *vs.* none	/	/	/	0.644	0.339–1.225	0.180
Breast conservation + radical mastectomy + local lumpectomy *vs.* none	/	/	/	0.639	0.336–1.216	0.172
Neoadjuvant therapy (yes *vs.* no)	/	/	/	1.157	0.851–1.573	0.352
Treatment before metastasis		/	/			
Chemotherapy (yes *vs.* no)	/	/	/	1.638	0.786–3.215	0.188
Radiotherapy (yes *vs.* no)	/	/	/	1.206	0.887–1.639	0.232
Targeted therapy (yes *vs.* no)	/	/	/	0.632	0.327–1.223	0.174
Endocrine (yes *vs.* no)	/	/	/	1.428	0.952–2.140	0.085

OR, odds ratio; BM, body mass index; TNBC, triple-negative breast cancer.

**Table 3:** Multivariable logistic regression analysis for LM in breast cancer patients with *de novo* and recurrent distant metastasis.

Variable	Cohort A (N = 325) Patients with *de novo* distant metastasis			Cohort B (N = 903) Patients with recurrent distant metastasis		
	OR	95% CI	*P*-value	OR	95% CI	*P*-value
Age at metastasis (≥50 *vs.* <50 years)	0.635	0.361–1.117	0.115	/	/	/
ER status (positive *vs.* negative)	0.873	0.497–1.533	0.636	0.811	0.585–1.124	0.209
HER2 status (positive *vs.* negative)	2.845	1.618–5.000	0.000	1.689	1.217–2.343	0.002
HBV status (HbsAg+ *vs.* HbsAg−)	/	/	/	0.739	0.404–1.351	0.326
Histology (invasive lobular + other *vs.* invasive ductal)	/	/	/	0.349	0.147–0.831	0.017

OR, odds ratio.

### Survival analysis

**Figure 2:**
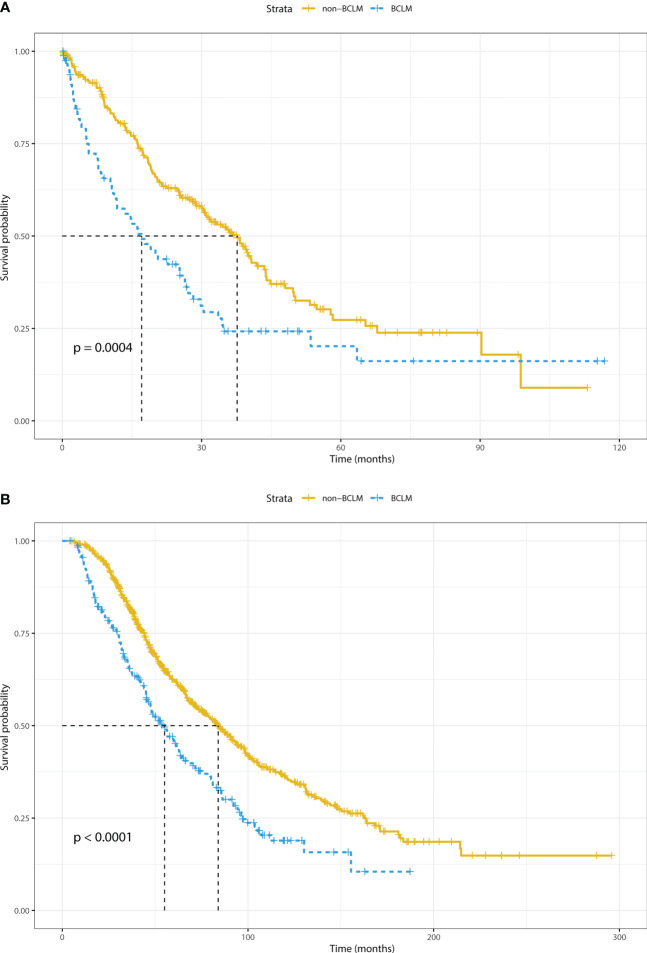
Overall survival of breast cancer liver metastasis (BCLM) and non-BCLM patients with **(A)** *de novo* and **(B)** relapsed distant metastasis. BCLM, breast cancer liver metastasis.

In total, 503 (55.7%) patients with recurrent MBC and 184 (56.6%) patients with *de novo* MBC died by the date of this analysis (17 September 2021). The median OS of the *de novo* and relapsed MBC populations was 30.4 and 80.1 months, respectively. **Figures 2A, B** show that BCLM patients had a significantly worse prognosis than non-BCLM patients both with *de novo* (17.1 vs. 37.7 months, *P* = 0.0004) and relapsed (47.6 *vs.* 84.0 months, *P* < 0.0001) MBC.

Univariable and multivariable Cox proportional hazard regression analyses were performed to assess the prognostic factors of BCLM patients. According to the univariable analysis (**Table 4**), the following variables were incorporated into the multivariable regression analysis: age at diagnosis, number of gravidity, HER2 status, primary T stage, bone metastasis, and lung/pleura metastasis for cohort A; and age at diagnosis, laterality, menopausal status, ER status, PR status, Ki67 level, nuclear grade, primary T stage, and history of neoadjuvant therapy and breast surgery for cohort B. As shown in **Table 5**, only the primary T stage (T4 *vs.* T1-3, HR = 2.128, 95% CI = 1.030-4.400, *P* = 0.042) was significantly associated with increased mortality for BCLM patients in cohort A. Regarding cohort B, Ki67 level (Ki67≥20% *vs.* Ki67 <20%, HR = 1.716, 95% CI = 1.009–2.918, *P* = 0.046) significantly increased mortality risk and breast surgery (yes *vs.* no, HR = 0.044, 95% CI = 0.005-0.415, *P* = 0.006) significantly reduced the mortality risk of BCLM patients.

**Table 4:** Univariable Cox regression analysis of overall survival (OS) in breast cancer patients with LM.

Variable	Cohort A (N = 81) Patients with *de novo* liver metastasis			Cohort B (N = 226) Patients with recurrent liver metastasis		
	HR	95% CI	*P*-value	HR	95% CI	*P*-value
Age at diagnosis (≥50 *vs.* <50 years)	1.569	0.919–2.680	0.099	1.731	1.238–2.419	0.001
Age at metastasis (≥55 *vs.* <55 years)	1.489	0.855–2.594	0.159	1.384	0.979–1.956	0.066
Laterality (right *vs.* left)	1.259	0.741–2.141	0.394	0.682	0.489–0.952	0.024
Menopausal status (post- *vs.* pre-)	1.158	0.683–1.964	0.586	1.534	1.092–2.153	0.014
Menarche (≥15 *vs.* ≤14 years)	1.483	0.842–2.610	0.172	0.780	0.532–1.144	0.204
Number of gravidity (≥3 *vs.* ≤2)	0.603	0.350–1.040	0.069	0.838	0.603–1.166	0.295
Number of parity (≥2 *vs.* ≤1)	1.350	0.978–2.285	0.263	1.327	0.955–1.844	0.092
BMI (>25 *vs.* ≤25)	0.910	0.522–1.586	0.740	0.831	0.598–1.154	0.268
Family history of cancer (yes *vs.* no)	1.040	0.536–2.016	0.908	0.715	0.450–1.135	0.155
HBV status (HbsAg+ *vs.* HbsAg−)	0.374	0.090–1.551	0.175	0.879	0.446–1.734	0.711
Histology (invasive lobular + other *vs.* invasive ductal)	1.147	0.358–3.681	0.817	0.970	0.393–2.394	0.948
ER status (positive *vs.* negative)	0.940	0.554–1.595	0.819	0.508	0.364–0.709	0.000
PR status (positive *vs.* negative)	1.025	0.593–1.772	0.931	0.464	0.329–0.656	0.000
HER2 status (positive *vs.* negative)	0.576	0.339–0.977	0.041	1.037	0.742–1.450	0.831
Ki67 (Ki67≥20% *vs.* Ki67 <20%)	1.002	0.531–1.889	0.996	1.950	1.272–2.987	0.002
Molecular subtypes						
HER2 positive *vs.* HR+HER2−	0.727	0.404–1.308	0.287	1.443	0.984–2.117	0.061
TNBC *vs.* HR+HER2−	2.725	1.225–6.060	0.014	2.805	1.785–4.409	0.000
HER2 positive *vs.* TNBC	0.766	0.494–1.187	0.233	0.613	0.370–1.025	0.057
Nuclear grade (III *vs.* I–II)	/	/	/	1.547	0.998–2.399	0.051
Primary T stage (T4 *vs.* T1–3)	2.604	1.511–4.489	0.001	2.180	1.382–3.438	0.001
Regional N stage (N1–3 *vs.* N0)	0.953	0.450–2.018	0.901	1.211	0.828–1.772	0.324
Breast surgery	/	/	/			
Breast surgery *vs.* none	/	/	/	0.254	0.138–0.467	0.000
Radical mastectomy *vs.* none	/	/	/	0.256	0.139–0.471	0.000
Breast conservation *vs.* none	/	/	/	0.814	0.101–6.535	0.847
Local lumpectomy *vs.* none	/	/	/	0.040	0.000–175.382	0.451
Palliative surgery *vs.* none	/	/	/	0.031	0.000–47.162	0.352
Breast conservation + radical mastectomy *vs.* none	/	/	/	0.257	0.140–0.472	0.000
Breast conservation + radical mastectomy+ local lumpectomy *vs.* none	/	/	/	0.256	0.139–0.470	0.000
Neoadjuvant therapy (yes *vs.* no)	/	/	/	1.510	1.087–2.098	0.014
Treatment before metastasis		/	/			
Chemotherapy (yes *vs.* no)	/	/	/	1.206	0.445–3.269	0.712
Radiotherapy (yes *vs.* no)	/	/	/	0.953	0.683–1.328	0.775
Targeted therapy (yes *vs.* no)	/	/	/	0.405	0.162–1.013	0.053
Endocrine (yes *vs.* no)	/	/	/	0.738	0.462–1.179	0.204
Distant lymph node metastasis (yes *vs.* no)	1.412	0.807 - 2.472	0.227	1.198	0.821–1.750	0.349
Bone metastasis (yes *vs.* no)	1.605	0.948–2.718	0.078	0.840	0.597–1.181	0.315
Lung/Pleura metastasis (yes *vs.* no)	1.917	1.120–3.281	0.018	1.110	0.799–1.543	0.533
CNS metastasis (yes *vs.* no)	1.200	0.566–2.544	0.634	1.558	0.816–2.975	0.179

HR, hazard ratio; BMI, body mass index; TNBC, triple-negative breast cancer; CNS, central nervous system.

**Table 5:** Multivariable Cox regression analysis of OS in breast cancer patients with LM.

Variables	Cohort A (N = 81) Patients with *de novo* liver metastasis			Cohort B (N = 226) Patients with recurrent liver metastasis		
	HR	95% CI	*P*-value	HR	95% CI	*P-*value
Age at diagnosis (≥50 *vs.* <50 years)	1.373	0.771–2.443	0.281	1.211	0.567–2.585	0.622
Laterality (right *vs.* left)	/	/	/	0.717	0.453–1.135	0.156
Menopausal status (post- *vs.* pre-)	/	/	/	1.059	0.491–2.283	0.884
Number of gravidity (≥3 *vs.* ≤2)	0.911	0.496–1.671	0.762	/	/	/
ER status (positive *vs.* negative)	/	/	/	0.598	0.329–1.088	0.092
PR status (positive *vs.* negative)	/	/	/	0.714	0.382–1.226	0.292
HER2 status (positive *vs.* negative)	0.633	0.360–1.113	0.112	/	/	/
Ki67 (Ki67≥20% *vs.* Ki67 <20%)	/	/	/	1.716	1.009–2.918	0.046
Nuclear grade (III *vs.* I–II)	/	/	/	1.028	0.611–1.731	0.916
Primary T stage (T4 *vs.* T1–3)	2.128	1.030–4.400	0.042	1.690	0.760–3.759	0.198
Neoadjuvant therapy (yes *vs.* no)	/	/	/	0.923	0.568–1.500	0.745
Breast surgery (yes *vs.* no)	/	/	/	0.044	0.005–0.415	0.006
Bone metastasis (yes *vs.* no)	1.359	0.749–2.466	0.312	/	/	/
Lung/Pleura metastasis (yes *vs.* no)	0.926	0.459–1.869	0.830	/	/	/

HR, hazard ratio.

## Discussion

Distant metastasis is a common sequela of advanced tumor stage that suggests unfavorable outcomes for breast cancer patients. In the present study, LM occurred in approximately 25% of patients with MBC, and it was ranked after bone and lung/pleura metastasis, which was consistent with previous studies (12, 29, 30). However, compared to recent data from the China National Cancer Center and Fudan University Shanghai Cancer Center, the proportion of *de novo* MBC patients was higher (26.5% *vs.* 9.4%, 26.5% *vs.* 20.4%, and 17.6%, respectively) (29–31), and the frequencies of specific organ metastasis were also higher than those previously reported (29, 31). These differences may be attributed to the following factors: differences or imbalances in patient groups; inclusion and exclusion criteria for the study population; medical levels; and socioeconomic factors. These differences emphasize the importance of early diagnosis and standardized treatment.

Specific standard-of-care therapeutic strategies for BCLM are lacking (6). However, in addition to systemic therapies, including endocrine therapies, anti-HER2-targeted therapies and chemotherapy, local therapies have application prospects for the treatment of BCLM. It has been reported that liver resection (LR) has favorable clinical outcomes in a selective population of BCLM, especially in patients with isolated LM or oligometastatic disease (17). Radiofrequency ablation may be utilized in patients with BCLM who do not benefit from LR (18). In addition, radical radiation therapy, radioembolization, transarterial chemoembolization, and radioembolization can be used in the management of some cases with BCLM (6, 32, 33). There is growing evidence that the incorporation of local intervention with systemic therapy is the most promising scheme for BCLM treatment (19–21). Thus, risk factors that identify BCLM patients in a timely manner will help clinicians make appropriate medical decisions for these patients.

Several studies have investigated risk factors for the development of BCLM (22, 34), but the majority of the previous research has focused on a specific population or analyzed MBC patients as a whole population. To our knowledge, the present study is the first and largest study to focus on LM patients with *de novo* or recurrent MBC in China, representing different MBC populations. Importantly, we found that patients with *de novo* and recurrent MBC had both similar and different risk factors involving LM, indicating that the risk odds for LM should be distinguished and evaluated between *de novo* and relapsed MBC populations.

In concordance with previous studies (34–36), the present study demonstrated that younger patients were more susceptible to LM than elderly patients, especially in posttreatment MBC patients. Compared to elderly patients, younger patients usually have tumors with higher malignancy or more aggressive behavior, which may eventually result in a greater tendency for LM or other organs of distant metastases (36–40). Although there were differences in distant metastasis patterns between these two study cohorts, BCLM patients were more likely to have synchronous polymetastasis. Furthermore, patients with MFS ≤1 year had a higher risk of LM in patients with relapsed MBC. These factors are indicators of aggressive tumors and a poor prognosis (29, 41).

Breast cancer is stratified into different subtypes, namely, histology and molecular. Previous studies have noted clear organ-specific patterns of metastatic colonization that are unique to each subtype (6). In the present study, HER2+ breast cancers aggressively spread to the liver in the pretreatment and posttreatment MBC population. These findings were validated by univariable and multivariable logistic regression analyses. This relationship between HER2+ breast cancers and LM has also been observed in other population-based studies in addition to those utilizing SEER data (13, 30, 42–48). Anti-HER2 agents improve disease outcomes for HER2+ breast cancer patients. Although patients in the present study who received anti-HER2-targeted therapy were limited, the proportion of BCLM patients with bone metastasis and CNS metastasis decreased compared to *de novo* MBC patients. Moreover, the HER2 status was not a predictor for the prognosis of the *de novo* or relapsed MBC population according to the Cox proportional hazard regression analyses. Thus, these results suggested that treatment changes the malignant behavior of some tumors. Furthermore, IDC breast cancer is the most common pathological type, and invasive lobular carcinoma (ILC) accounts for approximately 10%. A previous study reported that IDC most frequently metastasizes to the bone, lung, and liver, whereas ILC preferentially metastasizes to the gastrointestinal tract (41). In the present study, IDC was an independent risk factor of LM for patients with relapsed MBC, which was consistent with previous studies (28, 42). However, this association was not found in patients with *de novo* MBC.

The median OS of BCLM patients in the *de novo* and recurrent MBC populations was 17.1 and 47.6 months, respectively, which was inferior to that of non-BCLM patients (median OS was 36.7 and 84.0 months in *de novo* and recurrent MBC populations, respectively). The survival of BCLM patients with *de novo* MBC in the present study was consistent with that reported in previous retrospective studies, ranging from 12 to 31.4 months (13, 30, 49, 50). In addition, the survival of patients with recurrent BCLM was more prolonged than that of *de novo* BCLM. These differences may be partly attributed to the distinction of different MBC populations. In the Cox proportional hazard regression analyses, the primary T4 stage significantly increased the mortality risk for BCLM patients in the *de novo* MBC population. Patients with a higher Ki67 level were associated with increased mortality risk, and breast surgery reduced the mortality risk of BCLM patients in the relapsed MBC population. These results are, in general, consistent with previous findings. Moreover, it is noteworthy that the OS of newly diagnosed MBC patients was significantly worse than that of relapsed MBC patients. This may boil down to the aggressive behavior of the newly diagnosed metastatic tumor and the early diagnosis and early treatment of the relapsed tumor. These results emphasized the importance of early screening, diagnosis, and prompt treatment.

The present study had several limitations. Firstly, the present study was a retrospective study at a single institution, resulting in potential referral bias, and some parameters had missing values. Primarily due to the long time span of this study, the mode of treatment also changed during the course of the study. Moreover, the palliative treatment regime also varied over time or limited to treatment conditions at that time. All these factors would affect the survival of patients. In the present study, we preliminary analyzed the effects of radiotherapy, chemotherapy, endocrine therapy, and targeted therapy before metastasis on the survival of patients but did not analyze the impact of palliative treatment on the prognosis for these patients. Therefore, it was difficult to analyze the accurate impact of a particular treatment on the outcome of patients with MBC due to the complexity and variability of the treatments. Moreover, we only analyzed patients who presented distant organ metastasis at the initial diagnosis of MBC, while patients who developed LM later in the course of the disease were not included, which may have led to incomplete patient information. Meanwhile, we also did not analyze the correlation of the liver distribution of lesions with the metastatic pattern of the liver, which should be further illustrated based on large-sample prospective clinical study research. Finally, other parameters (e.g., other clinicopathological and molecular biomarkers or biochemical factors) that were not included in the present study may provide valuable predictive information, which should be included in future studies.

## Conclusions

The present study provided insight into the incidence and prognosis of LM at the initial diagnosis of MBC, including treatment-naïve and posttreatment relapsed cases in China. We identified different risk and prognostic factors for the LM of breast cancer patients with *de novo* and relapsed distant metastasis. These parameters may help to identify MBC patients with a high risk of LM and evaluate the prognosis of BCLM patients.

## Data availability statement

The raw data supporting the conclusions of this article will be made available by the authors, without undue reservation.

## Ethics statement

The studies involving human participants were reviewed and approved by Chongqing University Cancer Hospital. Written informed consent for participation was not required for this studyin accordance with the national legislation and the institutional requirements.

## Author contributions

XZ, DT and NZ conceived and designed the study. NZ, YX, DT, QS, JW, YL, HL and XZ collected and analyzed the data. NZ wrote the manuscript. XZ and DT reviewed and edited the manuscript. All authors contributed to the article and approved the submitted version.
